# Editorial

**Published:** 1983

**Authors:** 


### EDITORIAL

'Scire est nescire, nisi id me scire alius sciret.'

With a sigh of relief, this journal, believed to be the oldest provincial medical journal with a continuous record of publication, has reached its Centenary year.

It was started in 1883, 9 years after the foundation of the Bristol Medico-Chirurgical Society in 1874. Greig Smith was the first editor and including the present incumbent there have been twelve. Between 1953 and 1963 it became *The Medical Journal of the South West,* but, realising that it had overreached itself, reverted to its former title. It has had its lean times, but even through two World wars it has kept going. Failure to produce our journal without inordinate delays has bedevilled our recent history. John Wright, the great Bristol firm of printers and publishers of medical and scientific books and journals, has now taken us on at the start of our second century. We dare to hope that this time we are in safe hands. We have caught up on our backlog and this number appears on time.

It is fitting that history should provide much of the material of this historical issue, and we are especially grateful to two authors who have responded magnificently to invitations to write historical articles. Professor Bruce Perry has written on 'Famous Bristol Doctors' and Dr. Henry Eastes has set down his memorable presidential address to the Cossham Medical Society on 'The Fleece Medical Society'–that little group of friends who met thrice yearly at the Fleece Inn at Rodborough, near Stroud, between 1788 and 1793 and gave to the world Vaccination, the first clinical description of Thyrotoxicosis, associated the syndrome of Angina Pectoris with Coronary artery disease and recognised that Mitral Valve disease was due to antecedent Rheumatic Fever. To meet they had to travel on horseback from Bath (30 miles), Ross-on-Wye (20 miles), Chipping Sodbury (18 miles), Berkeley (12 miles) and Gloucester (10 miles). What a story!

In these days of audio and video technology one may ask oneself if the written word is here to stay. Perhaps, at any rate among professional men, it has another century to run. However, one erstwhile mode of communication among medical men – the Latin language – has not survived the century since our journal was founded. On the cover of the first number of the journal and of every number until 1901, was the epigram of Lucilius (180–102 B.C.) which still could not be more appropriate. The exact meaning, it must be admitted, escaped your editor who consulted a legal friend for whom that language still has life. He translated it elegantly as:

'What I know remains unknown, unless by me to others shown.'

